# Recent Advancements in Photocatalytic Synthesis of Five Membered Nitrogen Heterocycles and Their Derivatives

**DOI:** 10.3390/molecules30173490

**Published:** 2025-08-25

**Authors:** Zeeshan Haider, Ravi Archana, Heongkyu Ju

**Affiliations:** 1 Department of Physics and Semiconductor Science, Gachon University, Seongnam-si 13120, Republic of Korea; zeeshanhaider@gachon.ac.kr (Z.H.); archanaravi205@gmail.com (R.A.); 2 Gachon Bionano Research Institute, Gachon University, Seongnam-si 13120, Republic of Korea

**Keywords:** photocatalysis, organic synthesis, nitrogen heterocycles, five membered ring, mechanistic insights

## Abstract

Photocatalytic synthesis of heterocycles has emerged as a versatile strategy in organic synthesis. Among various heterocycles, five membered heterocycles such as pyrroles, indoles and their derivatives have great significance based on their pharmaceutical applications. Diverse photocatalysts have shown great potential in synthesis of nitrogen heterocycles either through radical-based mechanism or via energy transfer pathway. Compared to other synthesis routes, the photocatalytic approach offers unique advantages including green synthesis, one step reaction and approaching the challenging reaction to prepare nitrogen heterocycles. Tuning redox potential or tailoring triplet state energies of photocatalysts can play crucial role in selective and efficient synthesis of nitrogen heterocycles. In this review we have briefly covered the latest developments demonstrated for photocatalytic synthesis of five membered nitrogen heterocycles including pyrroles and indoles and their derivatives. We also discuss the existing challenges, bottlenecks and the future outlook in this field, aiming to advance photocatalytic strategies of producing five membered nitrogen heterocycles as valuable tools in modern synthetic chemistry.

## 1. Introduction

Photocatalysis is a light energy fueled catalytic approach for driving diverse chemical reactions. Ideally, utilizing solar energy for boosting chemical reactions enables an environment-friendly and sustainable route for chemical synthesis. The basic principle of photocatalysis is based on absorption of photons followed by generation, separation and transportation of charge carriers for surface redox reactions to take place [1]. Metal oxide-based semiconductors are one of the most attractive candidates for driving photocatalysis owing to unique features such as natural abundance, ease of fabrication and chemically stable structures [2,3,4,5,6,7]. In addition, organic semiconductors such as graphitic carbon nitride, (C_3_N_4_), carbon quantum dots, covalent organic frameworks and biomass-derived carbon have recently emerged as photocatalysts [8,9,10,11,12]. These organic semiconductors have frequently been applied for catalyzing various chemical reactions that produced valuable outcomes such as generation of hydrogen via solar energies, CO_2_ reduction to valuable chemicals, H_2_O_2_ production, biomass transformation, upcycling of microplastic and waste remediations [13,14,15,16,17,18,19,20]. Heterostructure formation has been found useful to boost photocatalytic activities [21,22,23,24].

**Figure 1:**
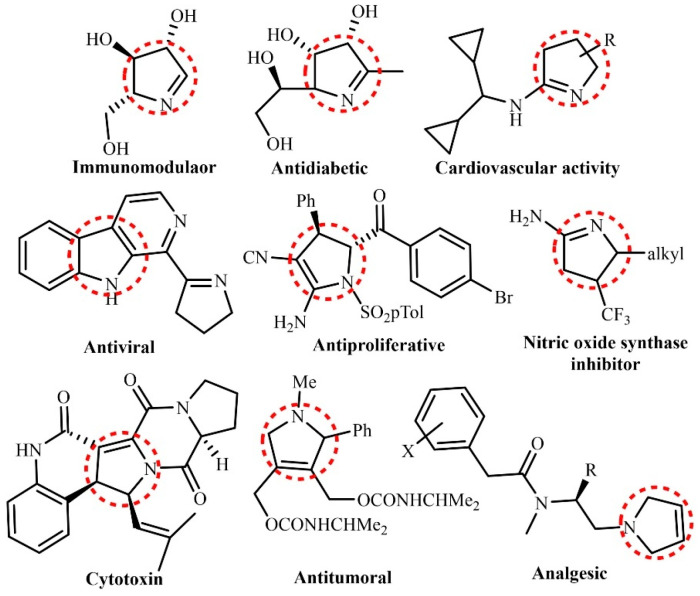
Biologically active pyrrolines [48]. Copyright 2025 Wiley.

In particular, photocatalysis has proven to play a vital role for organic transformations through hetero-cyclization reactions [25,26,27,28]. It is known that photocatalysis requires less stringent conditions for organic synthesis because it utilizes light energy to activate the photocatalyst, which can favor the occurrence of chemical reactions under mild conditions of temperature and pressure [29,30,31,32,33]. In other words, ability of photocatalysts to generate intermediate radical species without sophisticated conditions leads to facile synthesis [34,35,36,37]. For instance, this has led to extensive exploitation of photocatalytic formation of C-N and C-C bonds for synthesis of diverse heterocycles including indoles, quinolines and pyrroles [38,39,40,41,42,43]. These conversions can be realized in an environment-friendly way at less elevated temperatures without toxic reagents, which makes them favorable for green chemistry [44]. Heterocycles as common chemicals can also be applied in various applications including pharmaceutical, chemical and textiles industries. Integrating photocatalytic approach into green synthesis of heterocycles may hold the promise in the future synthetic chemistry for a large-scale production of chemicals [45,46]. Among various heterocycles, five membered nitrogen-containing heterocycles such as including pyrroles and indoles and their derivatives hold great significance due to their applications in pharmaceuticals (Figure 1). This review covers the recent trends in photocatalytic approaches for synthesis of nitrogen containing heterocycles including the relevant mechanistic pathways and the selectivity of synthesis. Limitations and challenges encountered in this field are also discussed, such as competing reactions to produce side products and limitations of lower product formation yield. Finally, the outlook is presented to drive the future progress for synthesizing nitrogen-containing heterocyclic organic synthesis.

**Figure 2:**
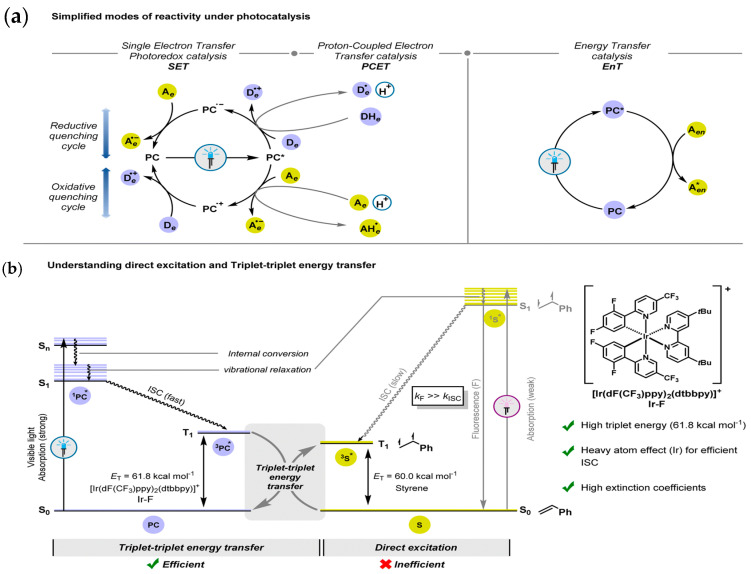
(**a**) Three different mechanistic approaches of photocatalysis. (**b**) Schematic representation (with an example of styrene and Ir–F) describing the unique aspects of TTEnT and comparison with direct excitation. * represents excited state. D: donor; A: acceptor; ISC: intersystem crossing; PC: photocatalyst; S: substrate; Sn: nth singlet state; T1: first triplet state [49]. Copyright 2023 Royal Society of Chemistry.

Light-induced cyclization in photocatalysis is governed by various pathways which can be categorized based on the production of intermediate species. The most common pathways are summarized as follows: (i) photoredox catalysis, (ii) energy transfer (EnT) and (iii) triplet–triplet energy transfer (TTEnT). Briefly, the photoredox pathway can proceed either through oxidative or reduction quenching cycles based on the substrate. During oxidative quenching cycle, the excited photocatalyst (PC*) can go through electron donation to an acceptor and PC^*^ itself is oxidized to radical cation (PC·^+^). Such an oxidized state of PC completes the cycle by accepting the electron form a donor [47]. Similarly, during the reductive quenching, phenomena take place by acceptance of electrons by the PC* from the substrate producing radical anion (PC·^−^). It is followed by the oxidation of PC·^+^ to the ground state of PC through interaction with electron acceptors [34]. An overview of various pathways involved in photocatalytic organic synthesis is summarized in Figure 2.

Such kinds of single electron transfer (SET) reaction promote the photocatalytic synthesis of nitrogen-containing heterocycles through the formation of radical intermediates [48,50]. A proton-coupled electron transfer involves the simultaneous transfer of electrons and protons during photocatalytic reaction. It is also commonly exploited for the synthesis of nitrogen-containing heterocycles [51]. Meanwhile, energy transfer (EnT) differs from the photoredox pathway in such a way that it does involve the transfer of excited state energy of the photocatalyst to the substrate instead of electron transfer. Such an exchange of energy between donor and acceptor species takes place through the phenomena of dexter energy transfer involving the orbital overlap of donor and acceptors. An EnT pathway is a potential strategy to drive cycloaddition reactions in organic synthesis [52]. The triplet–triplet energy transfer (TTEnT) mechanism is a kind of energy transfer pathway that is specific to the energy transfer from the triplet state of the photocatalyst to the triplet state of the substrate. This is a very attractive approach adopted for the synthesis of cycloaddition reactions. 

## 2. Light-Driven Synthesis of Heterocycles

### 2.1. Photocatalysts for Driving Heterocycle Synthesis

Traditionally metal and metal free, both classes of photocatalysts have been applied for the synesis of heterocycles. The choice of photocatalyst is greatly dependent upon the nature of reaction they are supposed to drive and the characteristics of substrates. On the basis of chemical composition, the photocatalysts commonly applied for the synthesis of heterocycles can be broadly classified into following categories. (i) Metal-free organic photocatalysts: various organic dyes such as Eosin Y, Rhodamine B and Rose Bengal have sufficient redox potential to drive SET-based reactions to execute C-H bond activation and cyclization reactions for heterocycles synthesis. Recently C_3_N_4_ has emerged as an attractive candidate for synthesis of heterocycles. (ii) Metal–organic-based photocatalyst: transition metal (Ru and Ir)-based polypyridyl complexes such as Ru[(bpy)_3_]^2+^, Ir(ppy)_3_ and covalent organic frameworks (COFs) have emerged as potential candidates for visible light synthesis of heterocycles. (iii) Metal oxide semiconductors, such as TiO_2_, can also effectively drive coupling and cyclization reactions during heterocycle synthesis [53,54,55].

**Figure 3:**
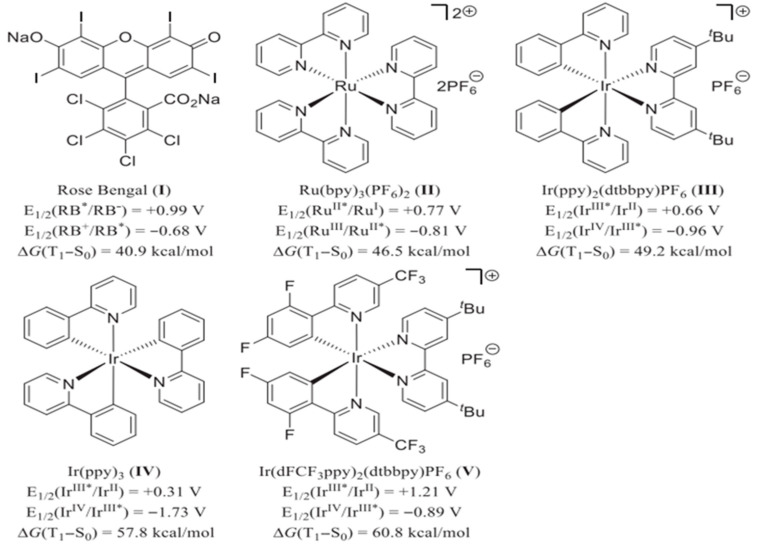
Commonly adopted photosensitizers along with oxidation/reduction potentials (vs. SCE) and triplet−singlet energy gaps [58]. E_1/2_ represents redox potentials. * represents excited state. Copyright 2019 American Chemical Society.

The redox potentials of the photocatalyst play key roles in determining their capability for driving specific reactions involved in heterocycle synthesis. Moreover, the energy gap between the singlet and triplet excited state of the photocatalyst is very important for effectively driving the energy transfer reactions, especially those involving intersystem crossing. Figure 3 summarizes the redox potential levels and energy gaps between the singlet and triplet excited states of the photocatalysts commonly applied for the synthesis and functionalization of heterocycles. Beyond the above-stated photocatalysts, intense efforts are devoted to engineering a novel photocatalyst for efficient and facile synthesis of heterocycles. For instance, the development of novel gold complexes [Au(SIPr)(Cbz)] (PhotAu **1**) and [Au(IPr)(Cbz)] (PhotAu **2**) prepared from n-heterocyclic carbene have shown excellent performance for indole synthesis [56,57]. They have shown potential to replace conventionally adopted Ir complexes for the synthesis of indoles and beyond.

### 2.2. Advancement in Heterocyclic Synthesis

Photoinduced synthesis is emerging as a potential strategy for C-C bond formation, cyclization reactions and cycloadditions reactions to prepare heterocycles. The latest research directions are increasingly focused on designing the new photocatalysts with appropriate redox potential and triplet state energies, reducing the multi-step reactions to one-pot synthesis and approaching radical-driven cascade reactions to prepare heterocycle products with higher yields. Adopting such directions, diverse synthesis strategies for a variety of heterocycles, particularly five membered nitrogen-containing compounds, have been developed, details of which are discussed below.

#### Five Membered Nitrogen-Containing Heterocycles

Five membered nitrogen containing heterocycles, particularly pyrroles and indoles, are frequently applied for medicinal applications. Several synthesis strategies exist for preparing these products, however, there are several limitations which restrict wider application of these routes. Therefore, there is growing interest in developing facile approaches to prepare five membered nitrogen heterocycles. A systematic overview of advancements made in the photocatalytic synthesis of such heterocycles is presented.

**Figure 4:**
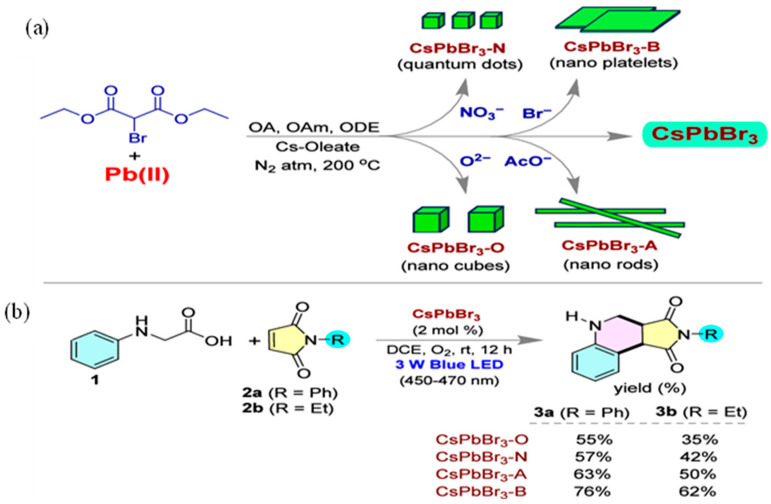
(**a**) By using diethyl 2-bromomalonate as the bromide precursor, we achieved the synthesis of CsPbBr_3_ perovskite NCs with varying dimensions through a hot injection method. (**b**) The radical cascade reaction of *N*-alkyl/aryl maleimide and *N*-phenyl glycine under blue LED light to yield an annulated product [59]. Copyright 2025 American Chemical Society.

**Figure 5:**
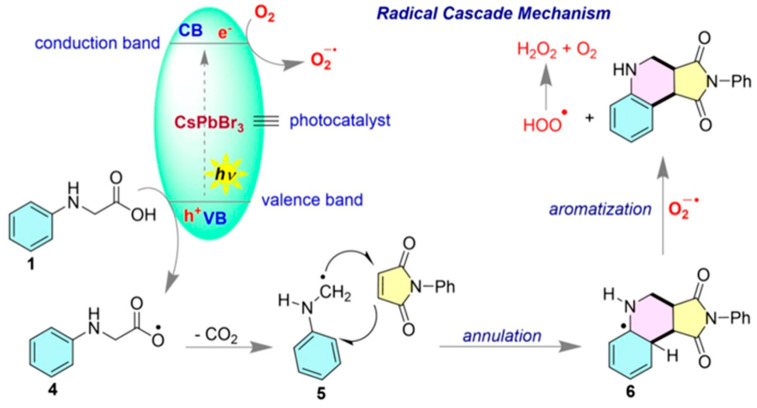
A plausible mechanism of the radical cascaded cyclization [59]. Copyright 2025 American Chemical Society.

Perovskite materials that have found applications for optoelectronic devices have great potential for advancing chemical conversion and catalyzing organic synthesis. Manna et al. have recently demonstrated the application of CsPbBr_3_ nanocrystals for radical-induced cascade cyclization of N-alkyl/arylmaleimide with N-phenyl glycine under blue LED illumination. It was found that the sizes and dimensions of nanoparticles greatly influenced the lifetime, a factor that influenced photocatalytic effects. CsPbBr_3_ nanoscale materials were prepared into nano cubes (CsPbBr_3_-O), nanorods (CsPbBr_3_-A), nano platelets (CsPbBr_3_-B) and quantum dots (CsPbBr_3_-N), as shown in Figure 4a. Of those, nanoplatelets have demonstrated the highest yield of chemical conversion for organic products, being ascribed to more effective capabilities for charge transfer under illumination. Figure 4b indicates that nanoplatelets have resulted in 76% yield for cyclo-condensation products with R=Ph as a reactant. In the case of R=Et, the nanoplate structure was still of the highest performance, resulting in 62% of the yield. The average lifetime analysis was observed as 6.11, 12.16, 14.67 and 15.13 ns for CsPbBr_3_-O, CsPbBr_3_-N, CsPbBr_3_-A and CsPbBr_3_-B, respectively. The longest lifetime observed for CsPbBr3-B has played an important role in enhancing the yield of cyclo-condensation reaction. Synthesis was suggested to proceed through the case of a radical cyclization pathway, as shown in Figure 5. According to the proposed mechanism, *N*-phenyl glycine is oxidized by valance band holes generated by the photocatalyst and radical intermediate was generated (4). The generated radical is transformed to another radical (5), resulting in the loss of CO_2_. This radical species reacts with *N*-arylmaleimide to produce the condensation radical product (6). In the last step it reacts with a superoxide radical produced by oxygen reduction by a conduction band electron to produce the final product 2-phenyl-3a,4,5,9b-tetrahydro-1H-pyrrolo [3,4-c]quinoline-1,3(2H)-dione. It reflects that both the valence band and conduction band actively participated to drive the overall cyclo-condensation reaction.

**Figure 6:**
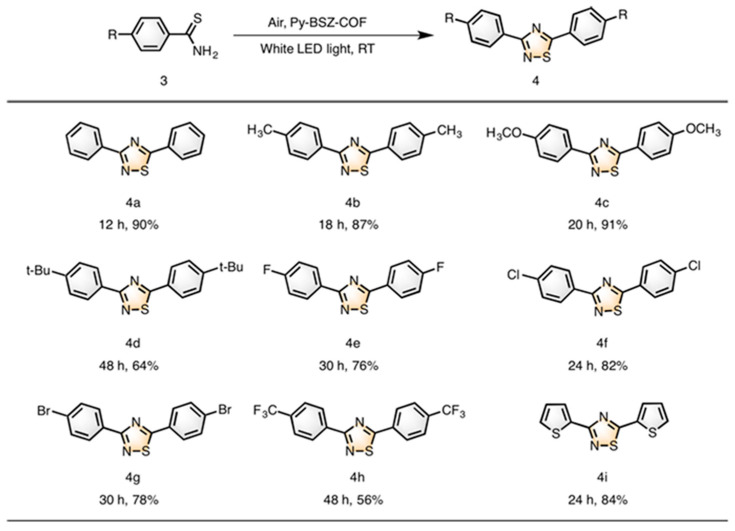
Scheme for cyclization of thioamide to 1,2,4 thiadiazole and description of diverse products (**4a**–**4i**) obtained by applying this reaction [60]. Copyright 2020 American Chemical Society.

Covalent organic frameworks (COFs) are emerging candidates for photocatalytic applications. Li et al. have recently demonstrated the application of a benzo thiadiazole-based COF to drive the photocatalytic oxidative coupling of amine and cyclization of thioamide [60]. The COF had a donor–acceptor structure that enhanced the efficiency for separation of charge carriers, resulting in a moderate-to-high yield of chemical reaction into cyclization during photocatalysis. It was found that the COF could act as a heterogenous platform for photocatalytic cyclization of diverse reagents into various products. The scheme of photocatalytic cyclization of thioamide is shown in Figure 6. It describes the general route for converting 4-R-thiobenzamide into 3,5-Bis(4-R)-1,2,4-thiadiazole. A variety of substituents (CH_3_, OCH_3_, t-butyl, F, Cl, Br, CF_3_) attached to thiobenzamide has been tested to prepare the diverse thiadiazole. Authors have suggested the following mechanism for the synthesis of thiadiazole. At first Py-BSZ-COF is excited to produce Py-BSZ-COF +, which reacts with thioamide and produces radical cation. The coupling of two radicals produces dimer, which undergoes cyclization. Superoxide radical anions produced by a reduction in molecular oxygen from the conduction band of photocatalyst facilitate the aromatization step to produce 1,2,4-thiadiazoles.

**Figure 7:**
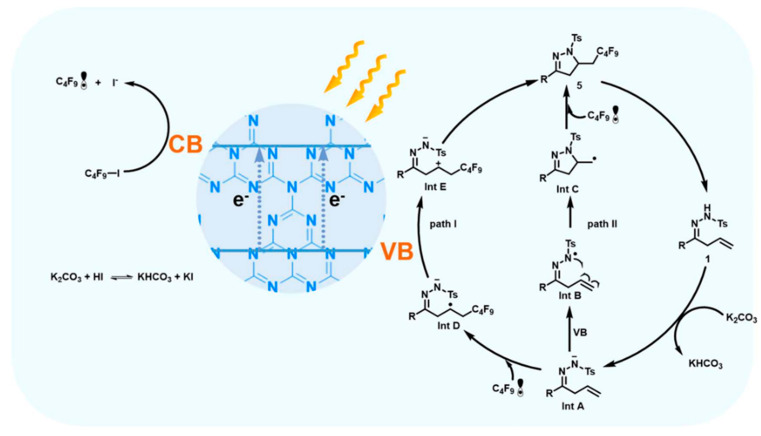
Proposed reaction mechanism for formation of radical-induced cyclization of hydrazone over carbon nitride [61]. Copyright 2022 Springer Nature.

Reaction conditions: thioamide (0.4 mmol), photocatalyst, Py-BSZ-COF (10 mg), 4 mL DMF, 1 atm air, and 15 W white LED bulb (11 mW cm^−2^); 3 = 4-R thiobenzamide, 4 = 3,5-Bis(4-R)-1,2,4-thiadiazole, 4a = 3,5-Diphenyl-1,2,4-thiadiazole, 4b = 3,5-Bis(4-methylphenyl)-1,2,4-thiadiazole, 4c = 3,5-Bis(4-methoxyphenyl)-1,2,4-thiadiazole, 4d = 3,5-Bis(4-trimethylphenyl)-1,2,4-thiadiazole, 4e = 3,5-Bis(4-fluorophenyl)-1,2,4-thiadiazole, 4f = 3,5-Bis(4-chlorophenyl)-1,2,4-thiadiazole, 4g = 3,5-Bis(4-bromophenyl)-1,2,4-thiadiazole, 4h = 3,5-Bis(4-trifluoromethylphenyl)-1,2,4-thiadiazole, 4i = 3,5-Bis(thiophen-2-yl)-1,2,4-thiadiazole.

g-C_3_N_4_ is a promising organic semiconductor owing to the ease of fabrication, highly specific surface area and capability to tailor its morphologies and functionalities. Yang et al. have highlighted the application of CN photocatalyst for cyclization of nitrogen-based radicals for the preparation of nitrogen heterocycles having pharmaceutical importance. In their work, they prepared 33 compounds using this approach explaining the effectiveness of CN-based catalyst for photocatalytic cyclization. It was shown that a positively charged CN photocatalyst based on higher affinity for anionic intermediates could boost the catalytic activity. The radical-based cyclization mechanism for synthesis of dihydropyrazole was shown in Figure 7. Anionic intermediate (Int A) produced by deprotonation was oxidized by valence band (VB) holes to produce nitrogen radical. It eventually underwent cyclization and a coupling reaction with C_4_F_9_ radical to produce the final product 5 3-(4-R-Phenyl)-5-(2,2,3,3,4,4,5,5,5-nonafluoropentyl)-1-tosyl-4,5-dihydro-1Hpyrazole. C_4_F_9_ radical production took place via conduction band electrons of CN.

TTEnT is another import mode emerging recently for driving organic transformations [62,63]. In this case, the photocatalyst undergoes intersystem crossing to reach the triple excited state, followed by the transfer to triple state energy to the suitable substrate as an acceptor. The energy transfer led the acceptor to its triplet state. There are energy losses due to I*_sc_*. The photocatalysts commonly applied for TTEnT should have high triple state energies. The emerging direction of applying TTEnT pathway in organic synthesis are discussed as follows.

Martynova et al. have developed a gold complex as a sensitizer for photocatalytic-induced cycloaddition reaction [56]. Their designed sensitizer is based on gold complexes with n-heterocyclic carbene aimed to replace the conventionally adopted Ir complexes. They have developed the following two photocatalysts, [Au(SIPr)(Cbz)] (PhotAu 1) and [Au(IPr)(Cbz)] (PhotAu 2). They have adopted these catalysts for [2 + 2] cycloaddition of diallyl ethers and N-tosylamides. It demonstrates an attractive strategy for preparing indole derivatives. It is notable that designed photocatalysts, [Au(SIPr)Cbz] and [Au(IPr)(Cbz)], have expressed higher triplet energies, which were found to be (66.6 and 66.3 kcal mol^−1^).

**Figure 8:**
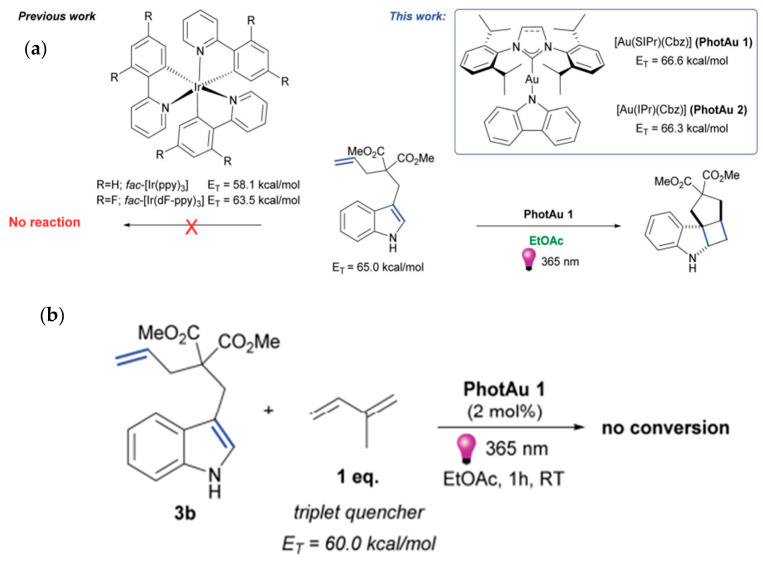
(**a**) The Ir photosensitizers and novel gold photosensitizers [Au(SIPr)(Cbz)] (PhotAu 1) and [Au(IPr)(Cbz)] (PhotAu 2) and the control experiment with a triplet quencher [56]. (**b**) reflect triplet energies of [Au(SIPr)Cbz] and [Au(IPr)(Cbz)]. Copyright 2022 Royal Chemical Society.

It has resulted to produce a higher yield of cycloaddition reaction. Moreover, the reaction time decreased to drive this reaction and conversions were proceeded in environmental benign approaching, avoiding the use of excess organic solvents adopted in conventional synthesis. The structure of designed photocatalysts and their ability to drive the cycloaddition reaction is shown in Figure 8. The Ir-based photocatalyst was previously found to be ineffective in driving this reaction. The Au-based complexes developed in this work have demonstrated effective photocatalysts to execute [2 + 2] cycloaddition reactions.

**Figure 9:**
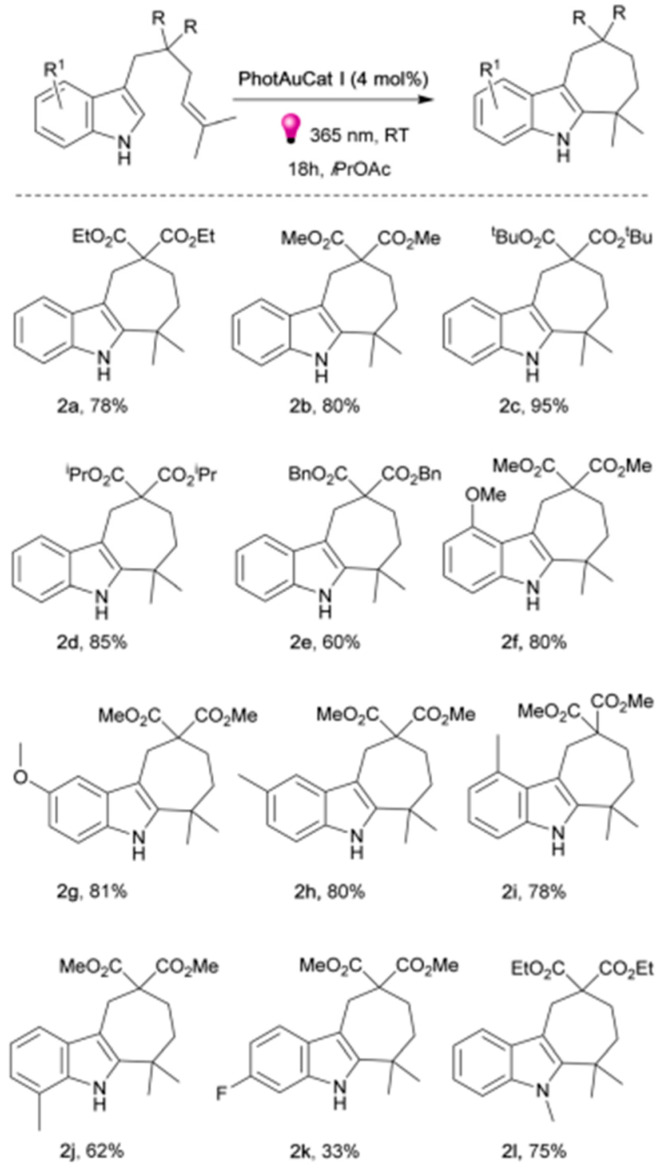
Substrate scope of indole derivatives. Unless otherwise noted, standard reaction conditions: 1a (0.10 mmol) and catalyst (0.004 mmol, 4 mol%) in solvent (2 mL) with 365 nm LEDs at rt for 18 h; (**2a**) = Diethyl dicarboxylate (2a) 6,6-dimethyl-6,7,8,10-tetrahydrocyclohepta[b]indole-9,9(5H), (**2b**) = Dimethyl 6,6-dimethyl-6,7,8,10-tetrahydrocyclohepta[b]indole-9,9(5H) S12 dicarboxylate, (**2c**) = Di-tert-butyl dicarboxylate 6,6-dimethyl-6,7,8,10-tetrahydrocyclohepta[b]indole-9,9(5H) Di-tert-butyl dicarboxylate, (**2d**) = Diisopropyl dicarboxylate 6,6-dimethyl-6,7,8,10-tetrahydrocyclohepta[b]indole-9,9(5H), (**2e**) = Dibenzyl dicarboxylate 6,6-dimethyl-6,7,8,10-tetrahydrocyclohepta[b]indole-9,9(5H), (**2f**) = Dimethyl 1-methoxy-6,6-dimethyl-6,7,8,10-tetrahydrocyclohepta[b]indole 9,9(5H) dicarboxylate, (**2g**) = Dimethyl 2-methoxy-6,6-dimethyl-6,7,8,10-tetrahydro cyclohepta [b]indole-9,9(5H) dicarboxylate, (**2h**) = Dimethyl 2,6,6-trimethyl-6,7,8,10-tetrahydrocyclohepta[b]indole-9,9(5H) dicarboxylate, (**2i**) = Dimethyl 1,6,6-trimethyl-6,7,8,10-tetrahydrocyclohepta[b]indole-9,9(5H) dicarboxylate, (**2j**) = Dimethyl 4,6,6-trimethyl-6,7,8,10-tetrahydrocyclohepta[b]indole-9,9(5H) dicarboxylate, (**2k**) = Dimethyl 3-fluoro-6,6-dimethyl-6,7,8,10-tetrahydrocyclohepta[b]indole-9,9(5H) dicarboxylate, (**2l**) = Diethyl 5,6,6-trimethyl-6,7,8,10-tetrahydrocyclohepta[b]indole-9,9(5H) dicarboxylate [57]. Copyright 2024 Royal Chemical Society.

On account of investigating the machoistic pathway, when isoprene was adopted as a triplet quencher no reaction was proceeded. It reflected that the cycloaddition reaction is proceeded through a TTEnT pathway. Zhao et al. have further extended the application of gold-based photocatalyst PhotAuCat I [Au(SIPr)(Cbz) for [2 + 2]-cycloaddition to prepare the variety of indole derivatives [57]. This intermolecular cycloaddition reaction proceeding through the TTEnT pathway demonstrates the high yield as shown in Figure 9. No product formation in the presence of the triplet quenching reagent has reflected the execution of the triplet energy transfer pathway. Energy transfer pathways have been tailored by combining the organic photosensitizer with perovskite materials to tune the band gap properties and regulating excited state properties [64].

**Figure 10:**
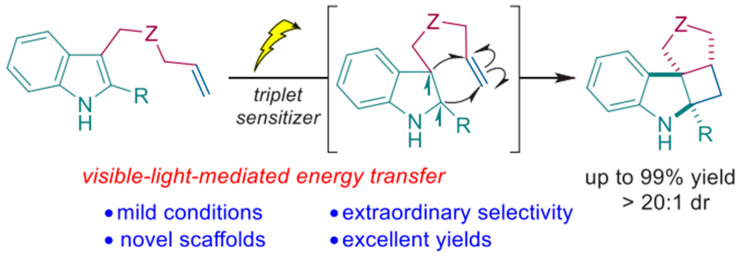
Triplet sensitizer-based intramolecular dearomatization of indole [58]. Copyright 2019 American Chemical Society.

Zhu et al. have demonstrated triplet sensitizer-based intramolecular dearomatization of indole with a high conversion yield, as shown in Figure 10 [58]. DFT calculations have been performed to unfold the reaction mechanism and find structure–activity relations.

**Figure 11:**
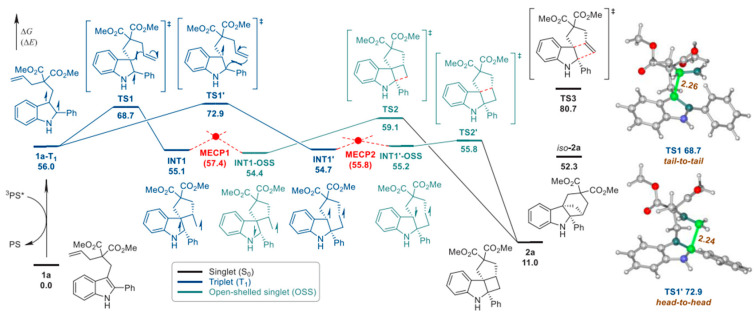
Energy profile of the visible-light-induced [2 + 2] cycloaddition reaction of indole derivatives, and the optimized structures of TS1 and TS1′. Calculated at the (U)B3LYP/6-31G(d,p) level of theory. The Gibbs free energies (ΔG) or electronic energies (ΔE, in parentheses) are in kcal/mol. ‡ represent TS. The bond distances are in angstrom [58]. * represents excited state. Copyright 2019 American Chemical Society.

As shown in Figure 11, smaller energy barrier 12.7 kcal/mol between TS1 relative to 1a-T1 has favored the execution of the cycloaddition reaction.

**Figure 12:**
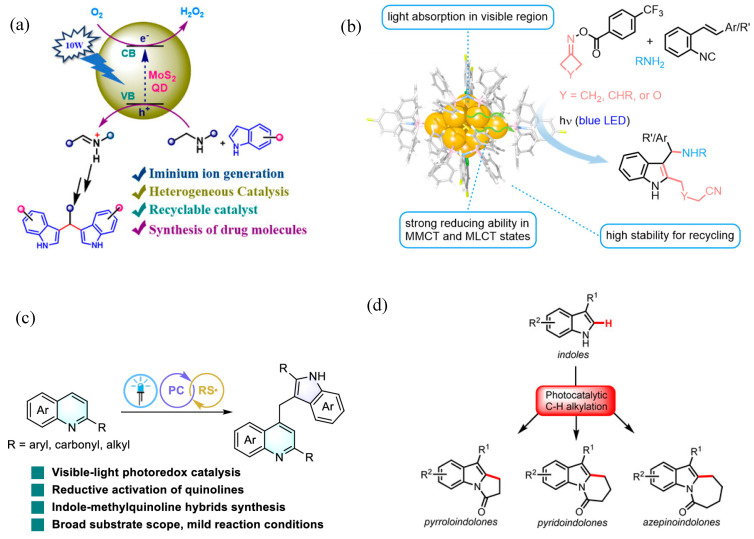
(**a**) Photocatalytic synthesis of Bis(indolyl)Methane derivatives [65]; copyright 2025, Wiley. (**b**) Johnson solid gold nanocluster for the synthesis of Fukuyama indole synthesis [66]; copyright 2025, American Chemical Society. (**c**) Scheme for synthesis of indole–methylquinoline hybrids [67]; copyright 2025, American Chemical Society. (**d**) Polycyclic indolones [68]; copyright 2020, Wiley.

Kayal et al. have demonstrated the application of MoS_2_ quantum dots (QDs) for the synthesis of bis(indolyl)methanes (BIMs). [65] This reaction proceeds through the formation of iminium ions as an intermediate species, which was converted to BIM. The synthesis scheme is shown in Figure 12a. This work unfolds the synthesis of bisindoline in a simplified approach under mild conditions. It can open new possibilities for the synthesis of indole-based compounds having biological significance to extend their applications in pharmaceuticals. Previous methods adopted for the synthesis of iminium ions as an intermediate face the challenges of competing side reactions, lower yield and being composed of multi-step reactions. However, the strategy unfolded in current findings overcome these challenges by generating iminium ions as stable intermediate in a facile approach for the preparation of BIM. While proposing the mechanism, authors have suggested that superoxide radical anion (O_2_^•−^) resulting from oxygen reduction reaction has played an important role to produce the iminium ions, which reacted with indoles to produce BIMs.

**Figure 13:**
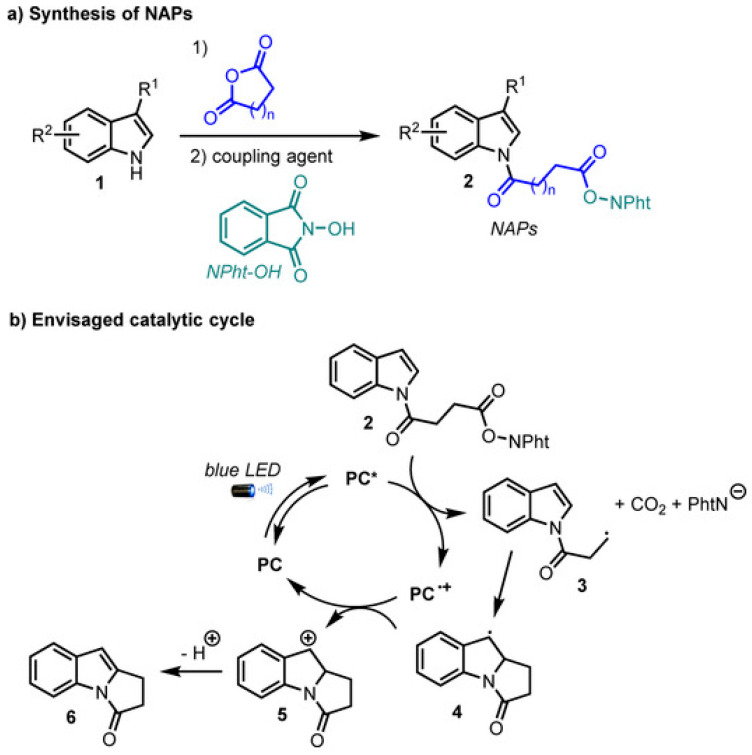
Catalytic cycle of the envisaged strategy [68]; * represents excited state. copyright 2020, Wiley.

Fukuyama indole synthesis is a versatile route for the synthesis of variety of indoles. Liu et al. have demonstrated the application of gold nanocluster for radical coupling to prepare indoles as shown in Figure 12b [66]. Gold nanocluster investigated in this work can facilitate electron transfer with O-acyl oximes. The significance of this work is to unfold the application of a metal nanocluster-based photocatalyst expanding the scope of Fukuyama indole synthesis. Gold nanocluster Au16 prepared in this work under blue LED illumination has demonstrated effective cleavage of N–O bond in O-acyl oximes during indole synthesis. The PNP ligand was adopted during the preparation of the phosphine-protected gold nanocluster. This gold nanocluster has expressed the occurrence of both metal to ligand charge transfer and metal to metal charge transfer. It demonstrates an interesting contribution of exploiting metal cluster-based visible light synthesis of nitrogen-containing heterocycles. In another interesting finding, Dai et al. have highlighted the photocatalytic synthesis of indole derivatives using a fac-Ir(ppy)_3_ photocatalyst, as shown in Figure 12c [67]. Interconversion of the nitrogen heterocycle has been demonstrated with high yield. It proceeds through ring contraction of quinolines through radical-based mechanism. It has resulted in the formation of an indole–quinolines hybrid structure with broader scope and applicable for a variety of reactant variants. C2 substituted with various groups such as aryl, carbonyl and alkyl groups have reflected effective transformations. This work can open new possibilities for exploiting the conversion of quinoline radical anions into a variety of products. The quinoline radical anions were proposed to form through reductive activation involving SET. Passing through radical intermediate, rearrangement and ring contraction, indole–quinolines hybrid was produced. Saget et al. have demonstrated photocatalytic synthesis of polycyclic indolones using indoles [68]. It occurs through C-H activation and proceed through generation of alkyl radicals as intermediate and undergoes intramolecular cyclization steps to produce polycyclic indolones as shown in Figure 12d. This reaction is important to transform the economically available indoles into valuable indolones of higher commercial value. For this purpose, N-acyloxyphthalimides (NAPs) were applied as redox active esters and precursors for producing alkyl radical. The synthesis of NAPs using indole and cyclic anhydride is shown in Figure 13a. The mechanism of this reaction is given in Figure 13b. During photocatalytic reaction, NAPs can produce alkyl radical through SET, which undergoes through cyclic rearrangements, oxidation and protonation to produce the final polycyclic indolones. This strategy has facilitated the synthesis of variety of indolones.

**Figure 14:**
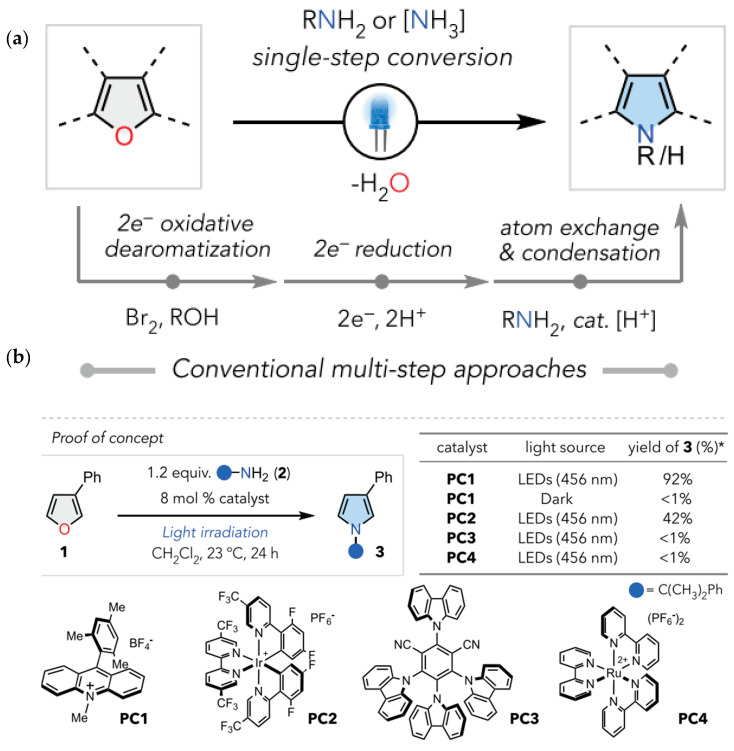
(**a**) Comparison of conventional multi-step approach and direct photocatalytic conversion of furan to pyrroles science [43], (**b**) (yield of different photocatalysts) * represents excited state; copyright 2024, American Association for the Advancement of Science.

Furan is a five-member heterocycle that contains oxygen as a heteroatom. The transformation of heterocycles form one to another could be complicated. For instance, transforming furan to pyrrole in conventionally adopted approaches may involve the complicated steps of dearomatization, substitution of the heteroatom and aromaticity recovery. In order to avoid such multi-step reactions, Kim et al. have demonstrated a simplified photocatalytic transformation of furan into pyrrole structures by substituting oxygen with nitrogen as shown in Figure 14a [43]. This important finding may open the new opportunities to transform naturally occurring furans into pyrroles. As proof-of-concept authors have presented transformation of g 3-phenylfuran (1) to cumylamine (2). Among various photocatalyst tested, PC1 has demonstrated excellent yield of product formation reaching 92% as shown in Figure 14b.

**Figure 15:**
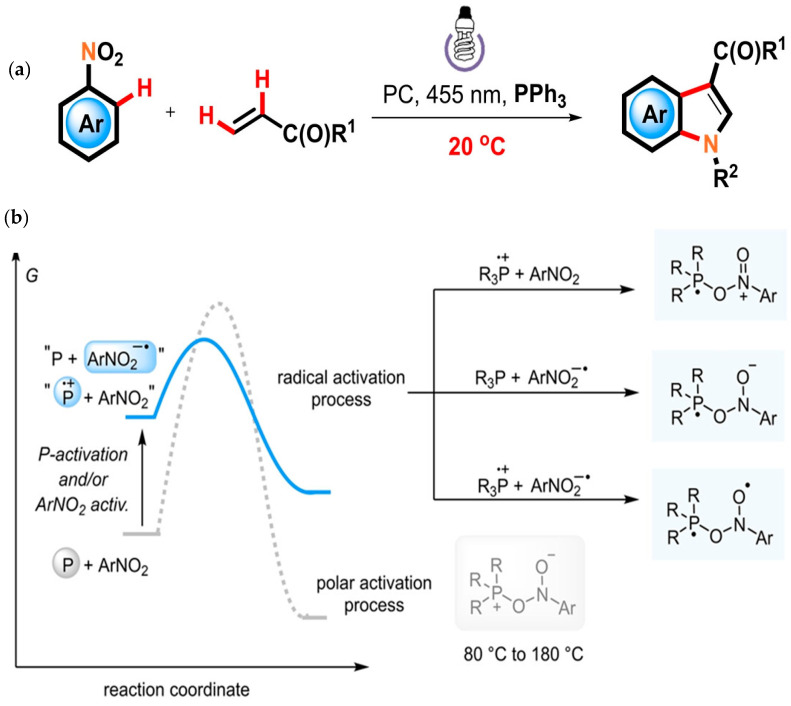
(**a**) Scheme for radical annulation process of nitroarenes and alkene and (**b**) organophosphorus-mediated polar activation process and suggested radical activation mode [69]; copyright 2024, Wiley.

Zhang et al. have highlighted triphenylphosphine (PPh3)-mediated radical-induced coupling of nitroarenes and alkenes to prepare indole derivatives at room temperature conditions as shown in Figure 15a [69]. Nitroarenes acted as a nitrogen source. For that purpose, organic phosphorus compounds have been used traditionally, which have high activation energy and need high temperatures to proceed with the reaction as shown in Figure 15b. The radical-induced activation of nitroarene by phosphines highlighted in this work has facilitated the synthesis of indole derivative at room temperature with good tolerance to functional groups in the presence of an Ir-based photocatalyst. It has resulted facile synthesis of C3 functionalized indoles. This work can open the new opportunities to prepare a variety of nitrogen-containing heterocycles under mild reaction conditions.

**Figure 16:**
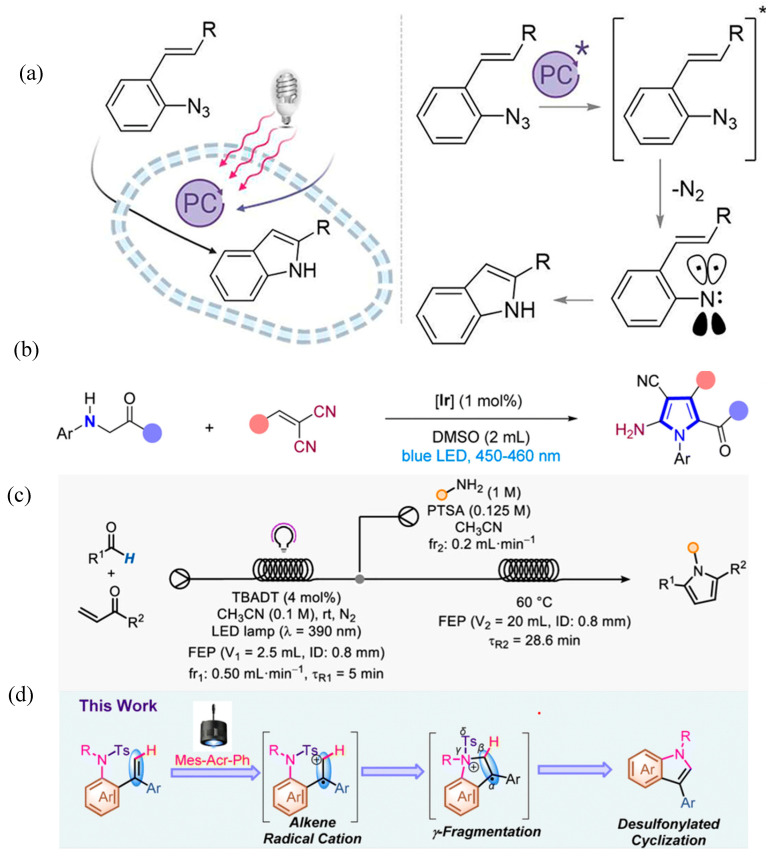
(**a**) Synthesis of indoles in cells and schematic illustration of mechanism [41]; copyright 2024, American Chemical Society, (**b**) copyright 2025 Royal Chemical Society [70]. (**c**) Scheme for photocatalyzed synthesis of pyrroles from glycinates [71]; copyright 2024, Wiley. (**d**) Scheme for synthesis of indole through desulfonylative C(sp2)-H-functionalization [72]; * represents excited state. copyright 2022, Royal Chemical Society.

Many indole derivatives have significance for pharmaceutical applications. In an interesting work, D’Avino et al. have demonstrated the synthesis of indoles within the cells [41]. For that purpose, living mammalian cells have been tested to prepare indoles using the photocatalytic approach. Such transformation has been performed under visible light illumination. Using ruthenium polypyridyl photocatalyst Ru(bpy)_3_][PF_6_]_2_, styryl aryl azides have been converted into 2-substituted indoles as shown in Figure 16a. The selection of low energy radiation such as visible light source for executing such reaction is particularly important so as to avoid the damage caused by irradiation to cells. Such a finding can be particularly useful for biomedical applications.

Sheng et al. have demonstrated green synthesis of poly-substituted pyrroles using the photocatalytic process [70]. They have adopted -aryl glycinates and 2-benzylidenemalononitrile for the synthesis as shown in Figure 16b. This process has shown higher functional group tolerance while producing pyrrole derivatives with higher yield. Previously reported methods for the synthesis of pyrrole derivatives through the [3 + 2] addition strategy involves the involvement of multi-step reactions and require the use of additional oxidizing agents. However, the strategy presented in this work is a facile and greener approach for the preparation of pyrrole derivatives. The approach presented in this work is based on producing radical intermediate from N-aryl glycine ester/amide, which undergoes cycloaddition reaction with benzyl dinitrile to produce the poly-substituted pyrroles. The synthesis approach has been tested with phenylglycine variants to produce pyrroles with diverse substituents. Sacchelli et al. have demonstrated a strategy for the synthesis of substituted pyrroles [71]. In this work photocatalyzed hydrogen atom transfer from aldehyde led to the production of 1,4-diketones, which were then subjected to Paal–Knorr or Hunsdiecker condensation to produce the pyrroles as shown in Figure 16c. It is challenging to prepare 1,4-diketones; in this work a facile approach has been demonstrated to prepare them. Formation of 1,4-diketones was proceeded through the formation of intermediate acyl radicals. Furthermore, integration of photocatalyzed synthesis of 1,4-diketones with PK or HC has opened new opportunities to advance the synthesis of substituted pyrroles. Beyond pyrroles, this strategy has also been extended for the preparation of other heterocycles such as thiophenes and cyclopentenones reflecting the diversity of this approach. Li et al. have suggested photocatalytic synthesis of N-substituted indoles from N-sulfonated arylalkene through γ-fragmentation without using the additional reagents (Figure 16d) [72]. Previously, various additives such as oxidants and bases were frequently applied for the synthesis of indoles. However, the facile approach presented in this work does not require the use of such reagents. This strategy has been designed by approaching simpler approach for desulfonylation. The intramolecular C-N bond formation strategy highlighted in this work through the desulfonylative process can open new opportunities to proceed for the synthesis of diverse heterocycles.

**Figure 17:**
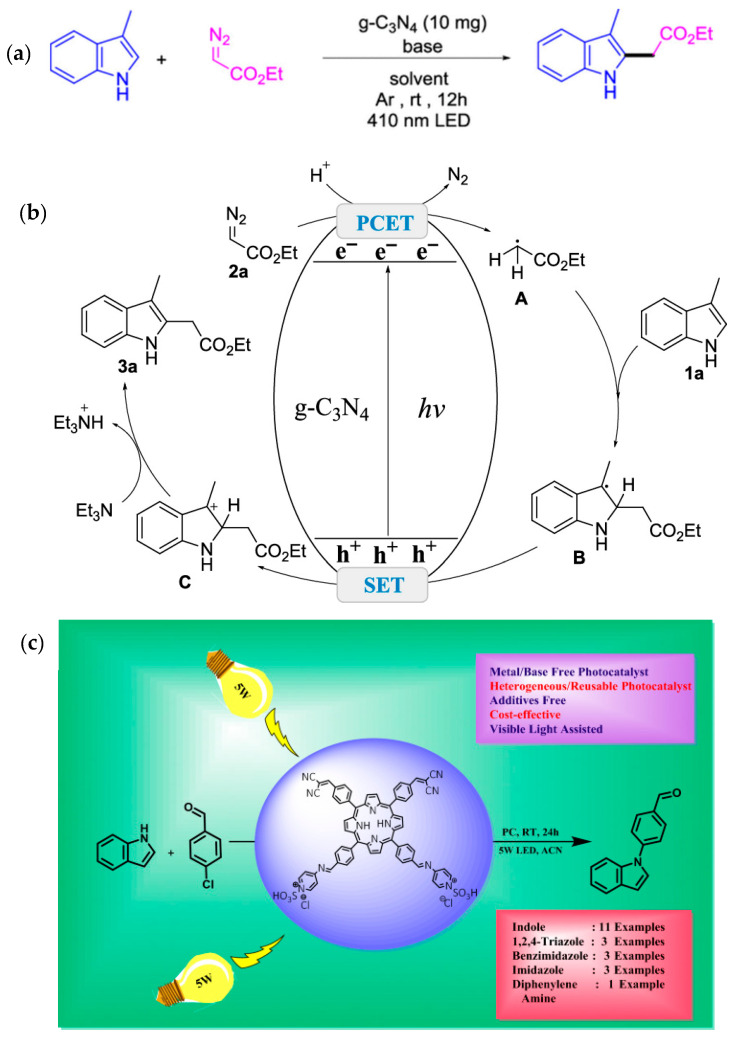
(**a**) C-H functionalization of indoles with diazo compounds and (**b**) corresponding mechanisms [73]; copyright 2023, American Chemical Society. (**c**) Synthesis scheme for fabrication of N-substituted indoles using aryl halides [74]; copyright 2024, Springer Nature.

Li et al. extended the application of C_3_N_4_ for the C-H functionalization of indoles with diazo reagents to prepare a variety of substituted indoles [73]. This presents a simplified approach induced by photocatalysis to prepare the indole derivatives. The photocatalyst showed stable behavior reflecting the potential for practical applications. The reaction proceeded through the PCET and SET pathways. In this work, 3-methylindole and ethyl diazoacetate were initially adopted to approach for the alkylation of the indole, as shown in Figure 17a. Later the utility of this synthesis scheme was extended by varying substrates to prepare diverse indole derivatives. The mechanism proposed for this reaction has been highlighted in Figure 17b. According to the suggested PCET pathway radical A was generated from ethyl diazoacetate. This intermediate radical reacts with indole 1a to produce another radical B, which undergoes oxidation through SET and deprotonation to produce the alkylated indole. The approach highlighted in this work demonstrated a simplified strategy for the synthesis of indole alkylation using a metal-free photocatalytic system. Patil et al. have extended the N-alkylation of indoles through formation of C-N bond using indole in the presence of a porphyrin-based photocatalyst (ILSAFPc) as shown in Figure 17c [74]. The indole substrate was reacted with aryl halides to functionalize N of the indoles with different aryl groups. Beyond synthesis of n functionalized indoles this strategy has also been extended for the preparation of derivatives of other diverse heterocycles such as imidazole and benzimidazole.

**Figure 18:**
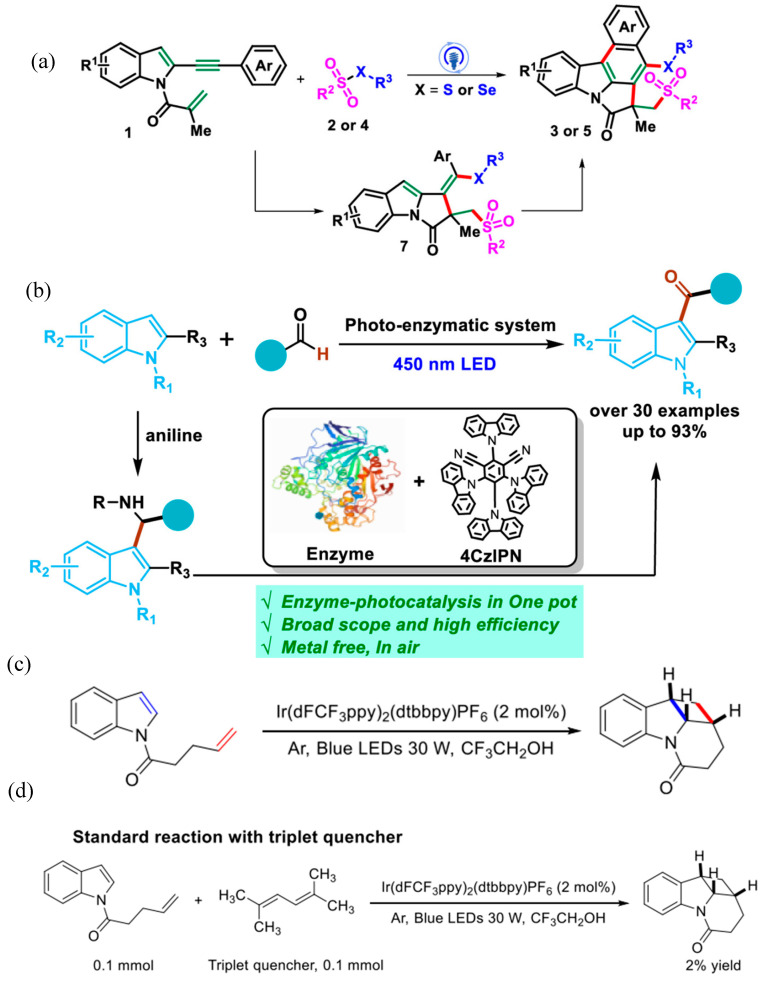
(**a**) Scheme for synthesis of photocatalytic thio/selenosulfonylation–bicyclization of indole [75]; copyright 2024, American Chemical Society. (**b**) Synthesis scheme for acylation of indoles using enzyme–photocatalyst approach [76]; copyright 2022, American Chemical Society. (**c**) Photocatalytic dearomative [2 + 2] cycloaddition of indole derivatives through energy transfer and (**d**) effect of triplet quencher [77]; copyright 2020, American Chemical Society.

The work highlighted in this work extends the application of a metal-free photocatalytic system for preparing diverse heterocycles. Zuo et al. have demonstrated bicyclization of indole-tethered 1,6-enynes. It has resulted in the synthesis of thio/selenosulfonylated benzo[c]pyrrolo [1,2,3-lm]carbazoles, as shown in Figure 18a [75]. In addition, it has resulted in one-step synthesis of two new rings induced by the photocatalytic process. It involves execution of several intermediate stages of homolytic bond cleavage, radical addition, radical coupling and a Mallory reaction. It shows good tolerance to functional groups and produced wide variants (over 50) by changing the substrate, reflecting the diversity of this process suited for practical applications. It demonstrates the possibility to extend the synthesis of various thiosulfonylated polycyclic carbazoles in a simplified strategy to introduce thio functionalities in nitrogen-containing heterocycles.

**Figure 19:**
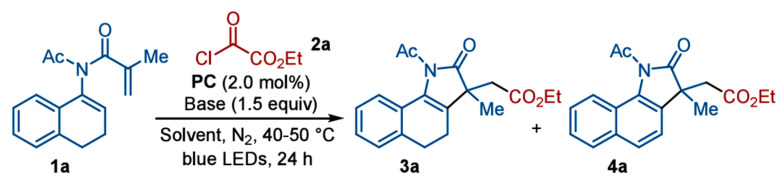
Scheme for synthesis of ester-containing pyrrolin-2-ones [78]; copyright 2025, American Chemical Society.

Another carbon–photocatalyst as a metal-free system has been introduced by Yu et al. for the synthesis of Acyl-substituted indoles using aldehydes, as shown in Figure 18b [76]. The synthesis strategy designed in this work has demonstrated synthesis of diverse 3-acyl substituted indoles with over 30 examples with higher yield around 93%. The designed strategy has the benefits of getting rid of harsh reaction conditions such as involving acid and metals as described in previous studies. The synergetic catalyzing effect of the enzyme and photocatalyst has facilitated to achieve the higher yield of indole acylation. Beyond tailoring radical-based reactions, [2 + 2] cycloaddition of indoles based on energy transfer route has been demonstrated by Zhang et al. as shown in Figure 18c [77]. It has been challenging to execute [2 + 2] cycloadditions of indole with olefins at N1 position. Such reactions are challenging due to unfavorable thermodynamics to drive such reactions. However, the strategy demonstrated in this work demonstrated execution of such additional reactions to produce polycyclic indoline. A hydrogen bond has promoted effective energy transfer to promote the [2 + 2] cycloaddition reaction. On account of investigating the reaction mechanism, when 2,5-dimethylhexa 2,4-diene was adopted as a triplet quencher the production yield was greatly reduced to 2% only as shown in Figure 18d. It reflects execution of energy transfer pathway for proceeding this cycloaddition reaction. Xiong et al. have demonstrated regioselective photocatalytic synthesis of ester-containing pyrrolin-2-ones using the Ir(ppy)_3_ photocatalyst through alkoxycarbonylation/cyclization under mild reaction conditions [78]; pyrrolin-2-ones due to their biological activities are an important class of organic compounds. The synthesis scheme for the fabrication of pyrrolin-2-ones is given in Figure 19. During synthesis, N-acetyl-N (3,4-dihydronaphthalen-1-yl)methacrylamide(1a) and ethyl chlorooxoacetate(2a) react in the presence of photocatalyst to produce ester-substitutedpyrrolin-2-one(3a) and dehydrogenative aromatization product(4a). Among various solvents tested, DMF has produced the best yield with 3a being 72% and 4a as negligible. Mechanistic investigations have revealed that the radical-based mechanism was involved in the formation of ester-substitutedpyrrolin-2-one.

**Figure 20:**
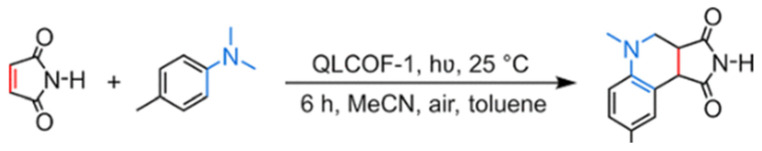
Scheme for synthesis of tetrahydroquinoline by annulation of maleimide and N,N,4-trimethylaniline [79]; copyright 2025, American Chemical Society.

Jun et al. in a recent finding have explored the imine-linked covalent organic framework (ILCOF) for C-H activation to prepare the quinolines as shown in Figure 20 [79]. ILCOF was then converted to Quinoline locked (QLCOF). They have demonstrated a superior response for C-H annulation exhibited by QLCOF compared to ILCOF. QLCOF has shown not only a higher yield for the annulated product, but it has also demonstrated higher catalytic stability when adopted for longer duration over 6 to 12 h. ILLCOF, on the other hand, has reflected poor stability after photocatalysis as observed through PXRD analysis before and after photocatalysis.

### 2.3. Comparison of Various Pathways

In summary, various pathways have been developed for the synthesis of oxygen and nitrogen-containing heterocycles using diverse photocatalysts including metal oxides, organic dyes, the metal–organic complex and carbon-based materials. Execution of various organic transformations are governed by various mechanisms. The synthesis pathways are mainly dependent on the photophysical properties of photocatalysts, substrate properties, thermodynamic considerations and characteristics of reaction medium. Each pathway driving the synthesis and functionalization of photocatalytic heterocycles have their own distinct features, merits and limitations. For instance, photoredox catalysis for synthesis of heterocycles has evolved as a potential strategy for synthesis of diverse compounds. The key advantage of this route is the generation of radical intermediates via SET, which can drive the versatile heterocycles. It also provides the advantages of higher tolerance of substrate functional groups. However, it requires very precise tuning of redox potentials of photocatalysts to achieve higher selectivity. This strategy often faces the limitations of producing side products due to occurrence of photoredox reactions in uncontrolled manner.

EnT specially offers unique advantages in photocatalytic cyclization reactions which on the other hand are difficult to achieve using photoredox catalysis. These features include the following benefits. Since it does not involve electron transfer during photocatalysis, it is useful in avoiding the side reactions in redox sensitive heterocycle synthesis. Because of the non-involvement of electrons during reactions this approach does not require the sacrificial electron donors and acceptors. Hence it simplifies the experimental conditions required for photocatalytic heterocycle synthesis. Moreover, this approach is more suitable for site selective cyclization due to the selective transfer of energy to specific sites.

TTEnT are very promising pathways for driving cycloaddition reactions. A variety of Ir- and Ru-based complexes having higher triplet state energy are frequently adopted for this strategy. However, in order to drive this pathway, the substrate should have appropriate triplet energy levels, which limits its applications to a wide variety of substrates. Since it involves ISC, it often faces the limitations of poor yield due to limited quantum yield for ISC energy transfer. Therefore, designing an efficient photocatalyst with higher ISC is essential for TTEnT-based photocatalytic synthesis of heterocycles. A brief comparison of various pathways for the synthesis of heterocycles is summarized in the following Table 1.

## 3. Challenges, Limitations and Future Outlook

Photocatalytic synthesis of pyrrole and indole derivatives has certain limitations and drawbacks in terms of selectivity, limited yield, catalyst stability and competing side reactions, and it still requires a deeper understanding of mechanistic pathways. Improvement in catalyst design and developing advanced tools for probing reaction mechanisms can boost greener synthesis of nitrogen-containing heterocycles. Photocatalytic transformations, being heterogenous systems, are facing the limitations of poor yield compared to homogenous reactions. It is further reduced by the poorly dispersed photocatalyst and light scattering effects. Photocatalysts applied for synthesis of nitrogen heterocycles also face the stability issues and experience degradation in efficiency upon prolong use. Higher recombination of photogenerated electron–hole pairs due to insufficient separation, especially for the case of photoredox catalysis, negatively affects the yield of desired products. Moreover, precise tuning of redox potentials for selectivity driving oxidative and reduction quenching remains a challenge. Metal oxide-based photocatalysts being naturally abundant materials are economically feasible to use, though most of the stable oxide materials absorb UV light due to wide band gap energies. Use of such high photon energy radiation is challenging to precisely control the selectivity of reactions and ensure the stability of produced organic products. Therefore, development of abundant photocatalysts having visible light absorption characteristics is an important point to consider. Photoredox catalysis is usually accompanied by the occurrence of competing side reactions.

Though organic photocatalysts that absorb visible light, they are facing the challenge of poor stability and undergo photodegradation easily. Precious metal-based catalysts have shown higher efficiency, but they do not lend themselves to large-scale application due to higher cost. Constraints in the deeper understanding of mechanisms especially for the case of EnT-based catalysis limit its application to broader fields. Use of sacrificial donors during photocatalytic processes makes the synthesis process more complex and requires rigorous efforts in product purification. Overcoming the above-mentioned limitations can significantly advance this research direction. In particular, improvement in photocatalyst design with enhanced photostability with tailored electronic structure and redox potential can greatly overcome the stability and selectivity issues. Improvements in mechanistic clarity through deeper understanding of intermediates species and excited state identifications would be useful to suppress the competitive side reactions. Tailored synthesis of novel photosensitizers that have high triplet energies are also needed. Moreover, it is highly desirable to develop water-soluble systems and replace the expensive iridium-based photocatalyst with abundant materials to make the synthesis economically viable and environmentally sound.

## 4. Conclusions

Photocatalysis has emerged as a promising approach to synthesize five membered nitrogen-containing heterocycles. As an alternative approach to conventional organic transformation, it offers unique advantages for green synthesis which avoids the use of toxic reagents. Execution of electron transfer and energy transfer pathways has facilitated the formation of five membered nitrogen-containing heterocycles under mild reaction conditions. The EnT and TTEnT pathways are particularly important for enhancing the selectivity of synthesis. However, despite the aforementioned advances in the photocatalysis, there are certain areas which necessitate further exploration such as developing novel photocatalysts with higher triplet energies, optimizing scalable synthesis conditions, enhancing functional group tolerance and minimizing the occurrence of competing side reactions. Attention should be paid to enhance the IS crossing efficiency to improve the yield of nitrogen heterocycles. Mechanisms of heterocycle formation, especially those involving multiple electron and proton transfer and formation of intermediate species should be investigated delicately. Development of novel schemes with different substrates will extend the scope of photocatalytic reactions to prepare pyrrole and indoles and their derivatives.

## Figures and Tables

**Table 1:** An overview of mechanistic routes involving photocatalytic synthesis of heterocycles.

Pathway	Key Mechanistic Aspects	Commonly Applied Photocatalysts	Specialty in Driving Heterocycles	Advantages	Limitations
Photoredox catalysis	Involving SET based radical intermediates	Semiconductor oxides, Organic dyes	C-C C-hetero bond formation	Mild reactions conditions Broader scope of application	Competing side reactions Need precise tuning of redox potentials
EnT	Transfer of Energy instead of electrons Can involve both singlet or triplet energy transfer	Complexes of Ir/Ru, organic dyes	[4 + 2] cycloadditions	Broader scope of application compared to TTEnT	Inappropriate for radical-induced cyclization
TTEnT	Triplet to triplet energy transfer	Complexes of Ir/Ru	[2 + 2] cycloadditions	Suitable for intermolecular cyclization Mild synthesis condition can drive the reaction Higher tolerance to functional groups	Lower efficiency for ISC Limited to substrate having matching triplet state energy levels Triplet state of photocatalyst should be higher than triplet state of substrate
